# THE HIDDEN TOLL OF INCARCERATION: EXPLORING THE LINK BETWEEN IMPRISONMENT HISTORIES AND PAIN AMONG OLDER ADULTS

**DOI:** 10.1093/geroni/igad104.1475

**Published:** 2023-12-21

**Authors:** Yulin Yang, Gabriel Lutz, Chixiang Chen, Raya Kheirbek

**Affiliations:** University of California, San Francisco, San Francsico, California, United States; University of Maryland School of Medicine, Baltimore, Maryland, United States; University of Maryland, Baltimore, Maryland, United States; University of Maryland School of Medicine, Baltimore, Maryland, United States

## Abstract

**Objective:**

Incarceration is linked to poor health outcomes across the life course. However, little is known whether and to what extent incarceration histories shape pain in later life. This study aims to examine the link between incarceration histories and pain outcomes among middle-aged and older adults in the United States.

**Methods:**

We used data from a nationally representative sample of community-dwelling adults aged 50+ in the 2012-2018 biennial waves of the US Health and Retirement Study (HRS) to estimate pain trajectory in U.S. older adults with and without a history of incarceration. We fit generalized estimating equation (GEE) models adjusting for socio-demographic covariates and early life experience to estimate the risk of reporting moderate-to-severe pain and related limitation.

**Results:**

We included 12,394 respondents aged 68.76 on average (SD= 10.34), 59% female, and 7% with incarceration histories (n= 870). Persons with incarceration histories have a greater risk of reporting moderate-to-severe pain (PR=1.38, 95% Confidence Interval [CI]: 1.24, 1.53) and pain-related limitation (PR=1.50, 95% CI: 1.34, 1.67) even after adjusting for socio-demographic covariates and early life experience.

**Discussion:**

In a nationally representative sample of older adults (with or without incarceration history), our study demonstrates an independent association between a history of incarceration and pain in later life. Our findings highlight the far-reaching impact of incarceration and the need for developing optimal pain management strategies to reduce the burden of disabling symptoms. Interventions should prioritize socioeconomically vulnerable groups, who may have the least access to pain treatment in later life.

